# Quasi-Static Mechanical Biomimetics Evaluation of Car Crash Dummy Skin

**DOI:** 10.3390/biomimetics9120762

**Published:** 2024-12-15

**Authors:** Yurun Li, Zhixin Liu, Cuiru Sun, Xiaoya Zheng, Guorui Du, Xiaoshuang Wang, Songchen Wang, Weidong Liu

**Affiliations:** 1 Department of Mechanical Engineering, Tianjin University, No. 135 Yaguan Road, Tianjin 300354, China; 2022201026@tju.edu.cn (Y.L.); carry_sun@tju.edu.cn (C.S.); 2022201208@tju.edu.cn (X.Z.); 2023201333@tju.edu.cn (G.D.); 2022201053@tju.edu.cn (X.W.); 2022201311@tju.edu.cn (S.W.); 2 China Automotive Technology and Research Center, Tianjin 300300, China; liuzhixin@catarc.ac.cn

**Keywords:** soft tissue mechanics, car crush dummy skin, viscoelastic properties, Ogden model

## Abstract

Accurate replication of soft tissue properties is essential for the development of car crash test dummy skin to ensure the precision of biomechanical injury data. However, the intricacy of multi-layer soft tissue poses challenges in standardizing the development and testing of dummy skin materials to emulate soft tissue properties. This study presents a comprehensive testing and analysis of the compressive mechanical properties of both single and multi-layered soft tissues and car crash dummy skin materials, aiming to enhance the biofidelity of dummy skin. We presented one-term Ogden hyperelastic models and generalized Maxwell viscoelastic models for single-layer and multi-layer soft tissues, as well as dummy skin materials. The comparative analysis results indicate that the existing dummy skin material fails to fully consider the strain-rate-dependent characteristic of soft tissue. Furthermore, dummy skin materials exhibited ~3 times shorter relaxation times and ~2–3 times lower stress decay rates compared to soft tissues, suggesting a less viscous nature. This study provides an accurate representation of the mechanics of soft tissue and dummy skin under quasi-static compressive loading. The findings are instrumental for the development of novel bionic skin materials or structures to more precisely replicate the biomechanical properties of soft tissues, thereby enhancing the accuracy and reliability of car crash test dummies.

## 1. Introduction

Car crash test dummies play a vital role in deciphering the human body’s biomechanical response to impacts during vehicular accidents. The accurate replication of human soft tissue properties is crucial for crafting the skin of these dummies, thereby ensuring the precision of biomechanical injury data. However, the skin of current crash test dummies, typically fabricated from rubber and polymers, falls short of mimicking the intricate biomechanical attributes of human skin. There is an urgent need for advancements in dummy design to enhance injury prediction accuracy, necessitating a quantitative mechanical equivalence analysis between dummy skin and soft tissue.

The mechanical properties of soft tissues are inherently linked to their structural complexity [1,2]. Despite the diversity in structure and function across biological soft tissues, the overall static or quasi-static mechanical properties are predominantly dictated by the tissue layers that constitute the majority of the composition [3]. The peripheral soft tissues enveloping the human body are primarily composed of skin, subcutaneous tissue (hypodermis or adipose tissue), and muscle tissue. Skin, as a complex multilayer material, exhibits nonlinear, anisotropic, viscoelastic, and nearly incompressible characteristics [4,5]. Subcutaneous adipose tissue, a soft connective tissue situated just beneath the dermis, is characterized by its low stiffness [6]. Muscles, integral to human movement, posture, and various functions, possess both active and passive mechanical properties [7]. The material properties of car crash test dummies are predominantly based on these three tissue types.

A variety of constitutive models have been proposed to describe the compressive properties of soft tissues. Shergold et al. [8] assessed the uniaxial compressive responses of silicone rubber and pig skin across a broad spectrum of strain rates (0.004–4000 s^−1^). They determined that a one-term Ogden strain energy density function sufficiently captures the constitutive behavior of pig skin under varying strain rates. John Z. Wu et al. [9]. selected pig skin samples for the compression test, and the results showed that the Ogden model could better simulate the experiment. Arch et al. [10]. Carried out a uniaxial tensile test on pig skin to fit parameters of various constitutive model types and found that the fitting error of Ogden model was more stable for different experimental results. Therefore, Kerstyn Comley [11] examined the response of adipose tissue over a wide range of strain rates and found that the Young’s modulus is almost insensitive to strain rate, with a value of 1 kPa at strain rates below 10 s^−1^. Adipose tissue demonstrates a nonlinear stress–strain response under diverse loading modes. The one-term Ogden model, used to model adipose tissue in various loading scenarios, performed comparably to other models, such as the isotropic Gasser–Ogden–Holzapfel (GOH) model and the hybrid neo-Hookean-exponential model [12,13]. Muscle tissue exhibits both passive and active mechanical properties [14,15]. In terms of passive behavior, Mo et al. [16] measured the compressive properties of skeletal muscles from different species under various loading rates and fiber orientations. They derived parameters for the one-term Ogden model and the three-term simplified viscoelastic quasi-linear viscoelastic (QLV) models, discovering that the average values of the Ogden model parameters for porcine muscles are closer to those of human muscles than those of bovine muscles. Van Loocke et al. [17] observed that the strain-dependent Young’s moduli provided a good fit to the experimental data up to 30% of quasi-static compressive strain. Although the Ogden model may not capture all the complex behaviors of muscle tissue, such as viscoelasticity and anisotropy, it is suitable for modeling strain-stiffening behavior [18]. The Ogden model can depict a broad range of nonlinear stress–strain behaviors, making it a primary candidate for studying the mechanical properties of skin, subcutaneous, and muscle tissues [19].

The material properties of full-thickness soft tissue are contributed by all its layers. Full-thickness soft tissue refers to the entire layer of soft tissue from the outermost layer of the skin to the innermost layer of muscle. However, studies on the mechanical properties of full-thickness tissue are scarce. In addition to hyperelastic properties, soft tissue also exhibits viscoelastic properties [20,21]. To develop adequate synthetic analogs, the compressive mechanical properties of these tissues are of particular interest due to the loading conditions typically encountered during a car crash. The skin of the car crash test dummy must correlate with the properties of soft tissue, including all layers. However, existing synthetic skin materials predominantly mimic only the skin layer [22,23]. A study of the mechanical properties of the layered structure for both soft tissue and dummy skin is essential to improve the design of car crush dummy skin.

This study aims to provide an in-depth investigation into the compression behavior of multi-layered soft tissues and car crash dummy skin. Through compressive uniaxial testing and comparison analysis, we elucidate the hyperelastic and viscoelastic properties of porcine soft tissue and dummy skin and propose how to enhance the biofidelity of dummy skin. The findings will offer comprehensive constitutive models for multi-type soft tissue and dummy skin, deepening our understanding of their biomechanics. Moreover, this study lays the groundwork for future studies on soft tissue and dummy skin during collisions, whether through simulation or experiment, with the ultimate goal of improving vehicle safety.

## 2. Materials and Methods

### 2.1. Preparation of Porcine Soft Tissue and Dummy Skin Specimens

**Figure 1:**
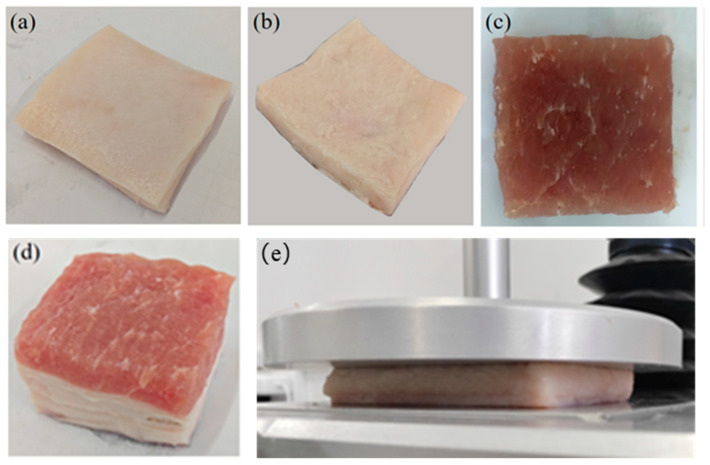
Photo of the porcine soft tissue specimens: (**a**) skin; (**b**) subcutaneous tissue; (**c**) muscle; (**d**) multi-layer soft tissue; (**e**) the skin sample under compression.

For our experiment, pork meat was procured from the Yingbin Vegetable Market in Jinnan District, Tianjin, China. The skin, subcutaneous tissue, and muscle specimens were meticulously prepared by dissecting each layer with a sharp scalpel blade and shaping them into blocks approximately 50 mm in length and width. Multi-layer soft tissue specimens, with dimensions similar in length and width to the single-layer ones, were also prepared. Representative images of some specimens are depicted in Figure 1. To ensure the reliability of our findings, at least 3 specimens of each soft tissue type were prepared for repetitive experiments.

**Figure 2:**
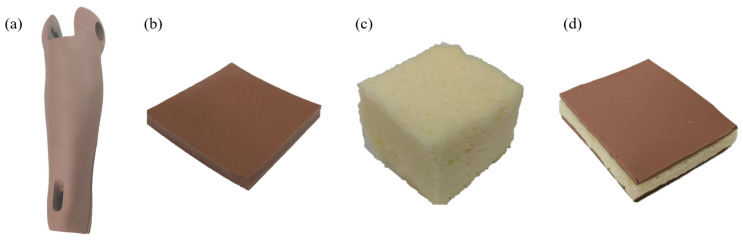
Photo of the dummy skin specimen: (**a**) the arm skin of a dummy; (**b**) rubber; (**c**) sponge; (**d**) multi-layer dummy skin material.

The car crash dummy skins were manufactured at China Automotive Technology and Research Center, Tianjin, China. The dummy skin material mainly consists of a rubber-like skin layer made of PVC material, a sponge-like filler layer formed by polyurethane foam, and an adhesive layer between the two. The production of dummy skin primarily employs injection-molding processes. The steps mainly include pouring the skin layer material mixture into the injection mold, allowing the skin layer to mature and form, then pouring the adhesive and centrifugally oscillating until it solidifies to form a uniform adhesive layer, followed by filling with polyurethane foam to create a uniform filler layer. Three distinct types of specimens were prepared from a dummy arm skin. As shown in Figure 2, these include specimens made from the rubber-like skin layer, the sponge-like filler material, and multi-layered composites that feature a central sponge layer sandwiched between rubber layers on the top and bottom.

### 2.2. Compression Test Protocol

Given that car crash dummies predominantly experience compressive forces during crash tests, we focused on conducting compressive experiments. The stable micro systems (TA. XT Plus Texture Analyzer, Lotun Science, Surrey, Godalming, UK) were utilized for uniaxial, unconstrained compression tests. The loading direction was aligned perpendicular to the skin surface. Two compression rates, 1.0 mm/s, and 0.5 mm/s, were applied, each reaching a maximum strain of 20%. To assess the viscoelastic properties, stress relaxation experiments were also performed. After, the specimen was deformed by 20%. The stress changes over time were recorded for 60 s when the strain was kept at 20%. Prior to mechanical testing, a 2.0 N pre-loading and unloading cycle was applied for 5 cycles to each specimen, ensuring uniform contact between the specimen and the compression plate and the reproducibility of the test results. The initial height of the specimen was recorded after pre-loading. Each soft tissue layer and dummy skin material was considered as an isotropic solid when determining the uniaxial compressive stress versus strain response. Consequently, the full-thickness specimens were regarded as transversely isotropic.

Due to the potential for permanent tissue deformation at high strain levels, each specimen was tested only once. However, to maintain experimental rigor, three specimens were prepared for each tissue type, resulting in three tests for each soft tissue type. This approach led to a total of 12 tests for each series of soft tissue tests, with 3 tests each for skin, subcutaneous tissue, muscle, and multi-layer tissue. For dummy skin samples, a 2.0 N preload was applied. The stress–strain curves of all the tests were fitted using the Ogden hyperelastic model [24]. In the uniaxial compression mode, with assumptions of isotropic, incompressible material, the engineering stress is formulated as follows [25]:

(1) $$\sigma =\frac{2\mu }{\alpha }(\lambda^{\alpha -1}-\lambda^{-\frac{1}{2}\alpha -1})$$

For compressible material, the model is expressed differently [26]:

(2) $$S_{1}^{in}=\mu_{0}\sum_{n=1}^{\infty }\mu_{n}\left(\lambda^{\alpha_{n}-1}-\lambda^{-0.5\alpha_{n}-1}\right)$$

In these Equations, *μ* and *μ_n_* represent the shear modulus, α and α_n_ are the strain hardening exponents, λ is the compression ratio, and S_1_^in^ denotes a stress component.

In order to evaluate the viscoelastic properties of both the soft tissue and the dummy skin, the stress relaxation test data were fitted by a generalized Maxwell model. The generalized Maxwell model consists of two Maxwell units in parallel and is expressed in Equation (3) [27]:

(3) $$\sigma \left(t\right)=E_{0}\varepsilon +E_{1}\varepsilon e^{-\frac{t}{\tau_{1}}}+E_{2}\varepsilon e^{-\frac{t}{\tau_{2}}}$$

where E_0_ is the initial elastic modulus; σ(t) is the stress at time t; E_i_ represents the elastic moduli; and τ_i_ represents the relaxation times of each Maxwell element. Through curve fittings, both the hyperelastic and viscoelastic material parameters were calculated.

To evaluate the accuracy of the constitutive model in fitting the test data, the average relative error was employed to assess the discrepancy between the test values and the model predictions. The average relative error, e, is calculated using the following formula:

(4) $$e=\frac{\sum_{i=1}^{m}\frac{\left|\sigma_{i}^{test}-\sigma_{i}^{fit}\right|}{\sigma_{i}^{test}}}{m}$$

where *m* is the number of measurement data; $\sigma_{i}^{test}$ is the stress value obtained from the test; $\sigma_{i}^{fit}$ is the stress value derived from fitting the constitutive equation.

### 2.3. Finite Element Analysis

Finite element simulation was carried out using COMSOL (6.2, Stockholm, Sweden) for stress analysis of the dummy skin. Single-layer and multi-layer models simulating the dummy skin material were created according to the specimens being measured. Each layer was assumed to be homogeneous, and the first-order Ogden hyperelastic constitutive model parameters were obtained from the experiments. The displacement in the vertical direction of the bottom was set as 0, and the top surface was applied a certain displacement. The model was meshed with hexahedral mesh elements. Transient analyses were performed.

## 3. Results

### 3.1. Hyperelastic Mechanical Properties of Soft Tissue and Dummy Skin

**Figure 3:**
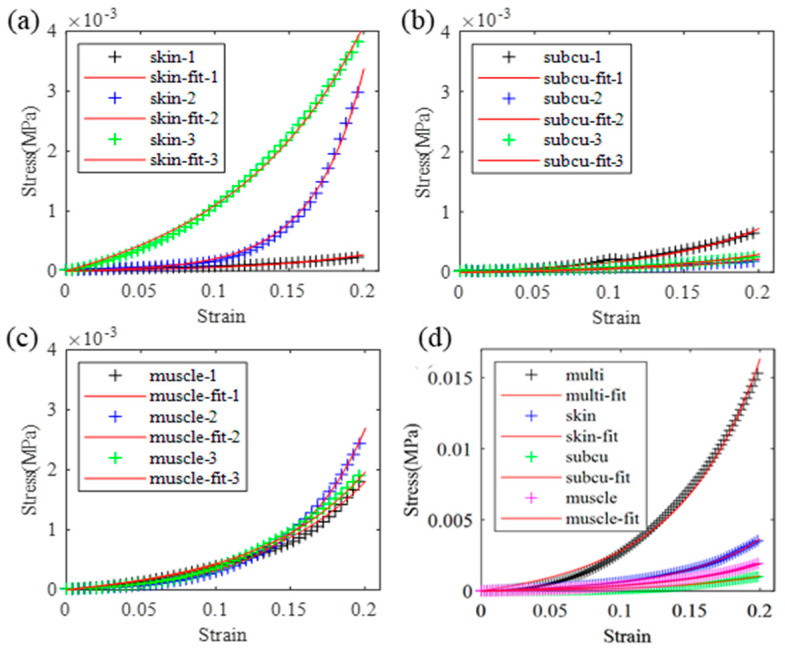
Compression test results of soft tissue subjected to 1.0 mm/s loading. Each type of tissue was tested three times, and the stress–strain curves were fitted with the one-term Ogden model. (**a**–**c**) Measured and fitted stress–strain curves of skin, subcutaneous, and muscle tissue specimens. (**d**) Average stress–strain curves of each tissue type and the multi-layer tissue sample. “subcu” represents “subcutaneous”, “multi” represents multi-layer tissue.

A total of twelve pork back tissue specimens, comprising skin, subcutaneous, muscle, and full-thickness multi-layer samples, with three of each type, were measured. The average dimensions of each specimen type are detailed in Table 1. The stress–strain curves obtained from the compression tests with 1 mm/s loading speed for the skin, subcutaneous, muscle, and multi-layer samples are presented in Figure 3. Each tissue type was subjected to three tests. It can be seen that the measurement results remain closely consistent, except for the curves in Figure 3a, where the bottom curve may have been affected by specimen irregularities. Consequently, only the upper two curves were considered for averaging purposes. The averaged stress–strain curves for each type of single-layer and the multi-layer samples are depicted in Figure 3d.

**Figure 4:**
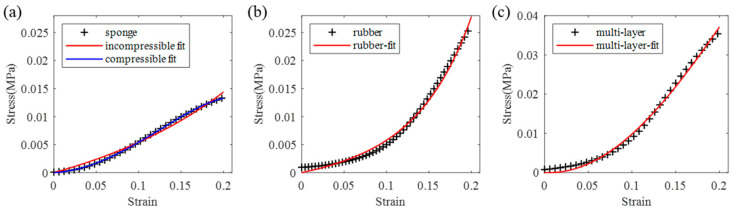
Compression test stress–strain curves of the dummy skin specimens with one-term Ogden model fitting curves in red: (**a**) curves of the sponge material with incompressible one-term Ogden model (red) and compressible multi-term Ogden model fitting curves (blue); (**b**) curves of the rubber specimen; (**c**) curves of the three-layer dummy skin specimen.

Multiple dummy skin specimens sized similarly to those detailed in Table 1 were tested, yielding stress–strain curves that exhibited similar patterns. Results of the dummy skin specimens shown in Figure 2 subjected to 1.0 mm/s compressive load are displayed in Figure 4. The dimensions of the dummy skin specimens are also listed in Table 1. Each specimen was tested once; thus, there is one curve for each type of the specimen. The curves were fitted using the one-term Ogden model.

By fitting the curves from Figure 3 and Figure 4 using the one-term Ogden model as per Equation (1), the parameters *μ* and a were determined and are presented in Table 1. The fitting error was calculated by Equation (4). It can be seen that the errors for soft tissue and rubber specimens are small (less than 6 × 10^−7^), which means they can be well described by the one-term Ogden model. Notably, the error is higher for sponge and multi-layer dummy skin specimens. However, the error can be significantly reduced when employing a three-term Ogden model with the assumption of compressible material, as illustrated in Figure 4a.

**Figure 5:**
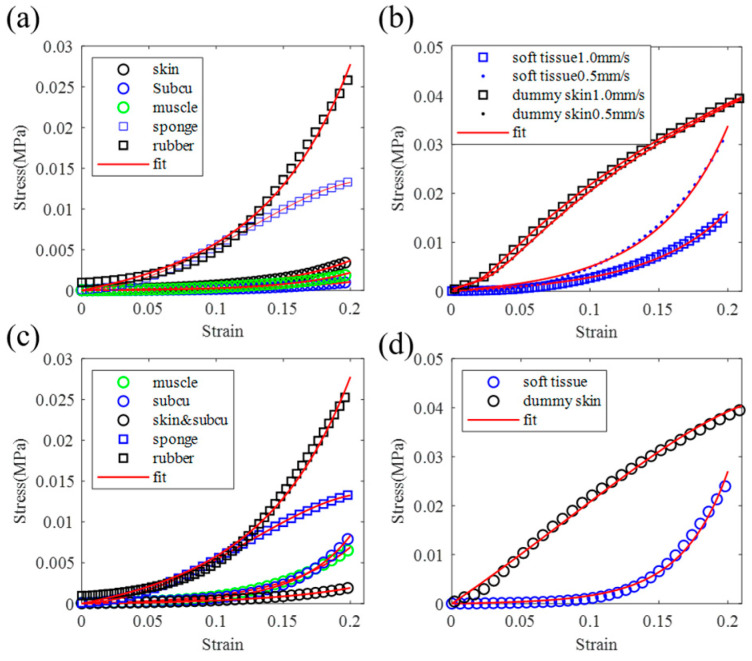
Stress–strain curves comparison between the soft tissue and dummy skin: (**a**) Single-layer dummy material with single-layer soft tissue from pig back; (**b**) Full-thickness multi-layer dummy skin with soft tissue from pig back under 1 mm/s and 0.5 mm/s loading rate; (**c**) Single layer dummy material with single-layer soft tissue from pig butt; (**d**) Full-thickness multi-layer dummy skin with soft tissue from pig butt under 1 mm/s. “subcu” represents “subcutaneous”.

To compare the mechanical properties of dummy skin and soft tissue, the dummy skin material was compared with different tissue types under different loading speeds. Single-layer dummy skin materials and soft tissue obtained from the pig back shown in Figure 3 and from the pig butt compressed with 1 mm/s loading speed are shown in Figure 5a and Figure 5c, respectively. The full-thickness multi-layer specimens of dummy skin and pig back soft tissue under 1 mm/s and 0.5 mm/s loading speed are compared in Figure 5b. The full-thickness multi-layer specimens of dummy skin and pig butt soft tissue under 1 mm/s loading speed are compared in Figure 5d. Figure 5 reveals the following: (1) The elastic modulus of soft tissue is considerably lower than that of dummy skin. In the lower-strain (<2.5%) phase, both materials exhibit characteristics of linear elasticity, and the elastic modulus is not affected by the loading rate. (2) After transitioning from the linear to the nonlinear phase, soft tissue shows strain hardening and loading-rate dependence, indicative of viscoelastic behavior. The dummy skin material, on the other hand, exhibits a characteristic of hardening followed by softening, which is nearly strain-rate-independent. (3) The area under the stress–strain curve represents the energy absorbed by the material during deformation. The areas under the curves from the bottom to the top in Figure 5b are E_1_ = 0.81 kJ, E_2_ = 1.44 kJ, E_3_ = 3.82 kJ, and E_4_ = 3.83 kJ. The energy absorption capacity of dummy skin material is similar under different loading rates, whereas the energy absorption capacity of soft tissue at a loading rate of 0.5 mm/s is 1.8 times of 1.0 mm/s loading rate, indicating its superior adaptability under dynamic loads.

### 3.2. Finite Element Analysis of Dummy Skin Specimen Under Compression

**Figure 6:**
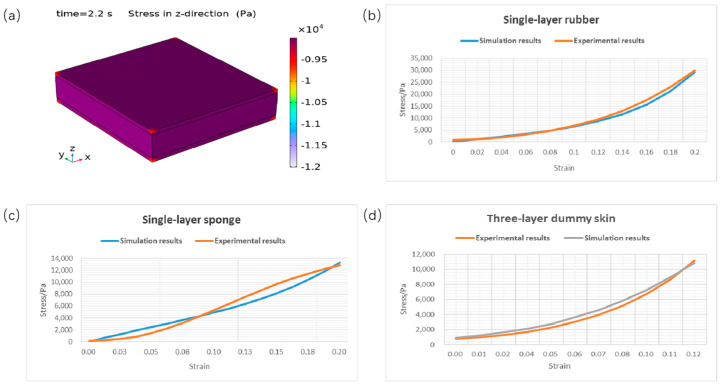
Finite element analysis of dummy skin. (**a**) Three-layer dummy skin model and z-direction stress distribution at 2.2 s; (**b**) Comparison of stress–strain curves of single-layer rubber specimen. (**c**) Comparison of stress–strain curves of single-layer sponge specimen. (**d**) Comparison of stress–strain curves of three-layer dummy skin.

Three models for the dummy skin specimen were created including one for the rubber layer, one for the sponge layer, and one multi-layered model according to the sizes listed in Table 1. The applied displacement on the top surface is 1 mm/s. The parameters of the one-term Ogden modes are also taken from Table 1. The simulation results are shown in Figure 6. It can be seen that the simulation results match with the experimental results.

### 3.3. Viscoelasticity Comparison Between Soft Tissue and Dummy Skin

**Figure 7:**
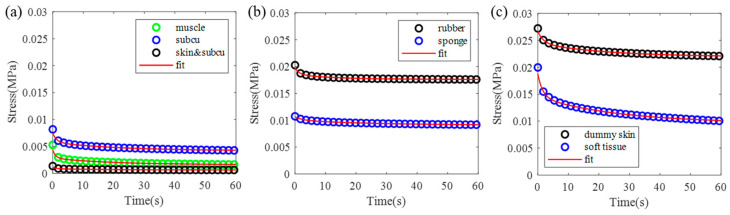
Stress relaxation measurement results of soft tissue and dummy skin with a constant strain of 20%. (**a**) Stress relaxation curves of soft tissue specimens, where “subcu” represents subcutaneous specimen, and “skin and subcu” represents a skin and subcutaneous bi-layer specimen. (**b**) Stress relaxation curves of rubber and sponge specimens. (**c**) Stress relaxation curves of full-thickness multi-layer dummy skin soft tissue specimens.

The soft tissue from pig butt and dummy skin specimens shown in Figure 5c,d were loaded to 20% strain for hyperelasticity measurement, then remained at the same strain for 60 s to evaluate their viscoelastic properties. The length and width of the specimens are similar to the samples listed in Table 1. The thickness of the specimens are listed in Table 2. Because it was difficult to separate the skin from the subcutaneous tissue, a skin and subcutaneous bi-layer specimen instead of a distinct skin layer was measured. The stress relaxation curves are depicted in Figure 7. To quantify the mechanical parameters, the Maxwell model expressed by Equation (3) was used for fitting these curves. Viscoelastic parameters, E_i_ and τ_i_, of all the specimens solved from curve fitting and fitting errors, e, calculated by Equation (4) are detailed in Table 2. The fitting results shown in Figure 7 and the fitting errors in the last column of Table 2 indicate the suitability of the two-units-generalized Maxwell model for modeling the viscoelastic properties of both soft tissue and dummy skin.

To further characterize the material behavior of the specimens, the initial stress and steady stress, defined as the stress change over time, are less than 5 × 10^−5^, the overall relaxation time, defined as the time when the steady stress is reached, and stress decay rate, defined as the change in the final stress at t = 60 s over the initial stress, were calculated and are detailed in Table 3.

According to the data in Table 2 and Table 3 and Figure 6, both dummy skin and soft tissues exhibit a decrease in stress over time, which is a common characteristic of viscoelastic material. The biological tissues—skin, subcutaneous tissue, and muscle—show a higher stress decay rate. Muscle tissue particularly exhibits a relaxation time of 27.6 s, which is more than 3 times longer than that of rubber and sponge specimens. The stress decay rate for the biological samples ranges from 50% to 65.4%, which is approximately 2–3 times higher than that observed in rubber and sponge specimens. The longer relaxation time of muscle tissue compared to subcutaneous and skin tissues suggests that muscle tissue requires more time to reach stress equilibrium. Additionally, the slope of the curves, and the stress decay rate listed in Table 3, indicate that the stress decay rate of muscle tissue is higher than that of subcutaneous and skin tissues. The composite tissue of skin, subcutaneous tissue, and muscle displays relaxation behavior that lies between that of skin alone and muscle alone. In contrast, dummy skin materials, including both rubber and sponge, exhibit a shorter relaxation time (8.4 s and 7.2 s, respectively) and a lower stress decay rate (~16%). This indicates that the dummy skin materials can maintain a higher stress level under strain, tending to exhibit more elastic behavior rather than viscous behavior. Notably, the relaxation time of the multi-layer dummy skin sample is 18 s, which is more than twice that of the individual rubber and sponge layers. Meanwhile, the stress decay rate of the multi-layer sample is 18.9%, indicating a relatively modest increase compared to the individual layers. These data indicate that biological tissue materials possess more pronounced viscoelastic characteristics in stress response, while dummy skin materials lean more towards elastic behavior. The high stress decay of muscle and soft tissue indicates a significant viscous component, allowing for the material to release stress over an extended period and demonstrating strong energy dissipation capabilities.

## 4. Discussion

The axial compressive mechanical properties of various soft tissue types and dummy skin materials were evaluated through experimental and numerical analyses. Our findings reveal that the peripheral soft tissues exhibit highly nonlinear, viscoelastic, and strain-rate-dependent characteristics. Dummy skin materials also demonstrate non-linear and viscoelastic properties, yet they fail to fully replicate the biomechanical behavior of soft tissue. The one-term Ogden incompressible hyperelastic model was found to accurately describe the behavior of the three main types of soft tissue, full-thickness multi-layer tissue, and rubber material under low strain rates (≤1 mm/s). However, the sponge material displays a distinct compressive behavior, as shown in Figure 4a, which diverges from that of the biological tissues. According to Figure 5, all tested biological tissues and rubber materials exhibit strain hardening, whereas sponges display a pattern of hardening followed by softening. The rubber–sponge–rubber composite material also shows a hardening–softening behavior, as depicted in Figure 5b. This suggests that the sponge material lacks sustained hardening ability during deformation, potentially affecting the structural stability and functional performance of the dummy.

**Figure 8:**
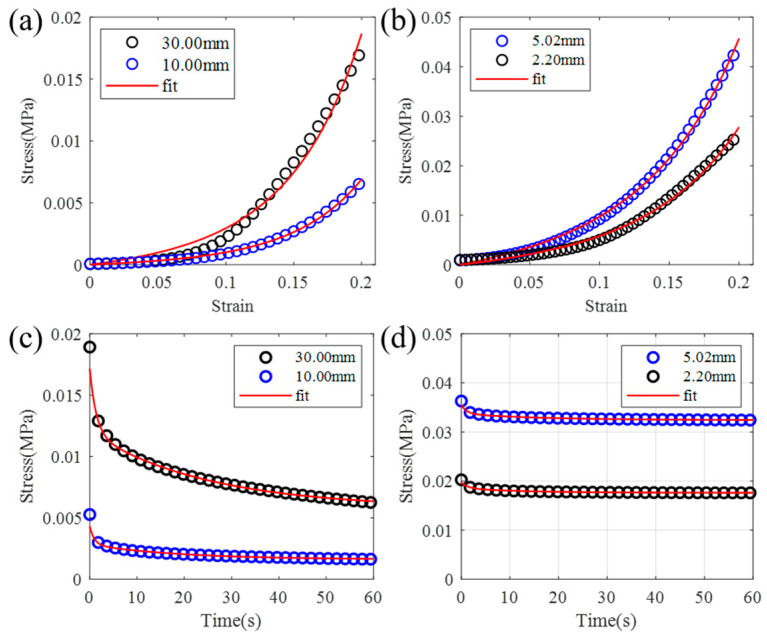
Influence of thickness to the mechanical properties of muscle and rubber material in dummy skin. (**a**) Stress–strain curves and (**c**) stress relaxation curves of muscle specimens with 10 mm and 30 mm thickness; (**b**) stress–strain curves and (**d**) stress relaxation curves of rubber specimens with 2.20 mm and 5.02 mm thickness.

The structure of biological tissue has an important influence on its function. The thickness of the soft tissue influences their mechanical properties. This is particularly true for muscle, where variations in thickness can affect volume changes during contraction and the muscle’s mechanical response [28]. Additionally, muscle thickness is related to its stress-bearing and distribution capabilities. We compared the compression responses of muscles and rubber materials with varying thicknesses under 1 mm/s of loading and stress relaxation at 20% strain, as shown in Figure 8. It can be seen that the hyperelastic and viscoelastic properties of the muscle and rubber material are all influenced by the thickness of the specimen. Thus, the thickness of the materials should be considered when designing the dummy skin, which also proposes the requirement for thickness-related constitutive models for both the multi-layer soft tissue and dummy skins in the future.

In this study, the density of the material was not taken into account. However, it is acknowledged that the density of a specimen can significantly influence its mechanical properties. Future research may explore the relationship between density and mechanical properties to better inform the design of dummy skin. The strains reported in this study were engineering strains, calculated based on the original dimensions of the samples. Changes in the cross-sectional area of the specimens were not considered during the compression tests. For scenarios involving large deformations, true strain calculations may be employed in future studies to offer a more precise depiction of the deformation behavior of both soft tissue and dummy skin.

There is no established standard for testing either soft tissue or car crash dummy skin. We referred to previous research on compression tests of soft tissues [29,30], ASTM D575—Standard Test Methods for Rubber Properties in Compression [31], and the Chinese national standard (GB5602-85)—Method for Multiple Compression Tests of Vulcanized Rubber [32]. Given the challenges associated with cutting regular shapes from soft tissue and the slippery nature of the subcutaneous layer, we opted to use cubical specimens. The results successfully demonstrate the quasi-static mechanical characteristics of both soft tissue and dummy skin. However, to enhance the robustness of our findings, future experiments may consider preparing cylindrical specimens.

**Figure 9:**
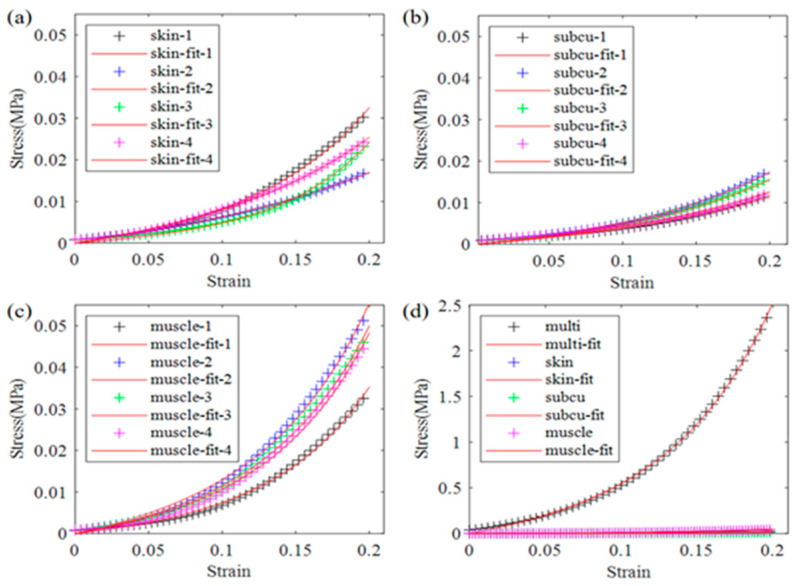
Compression stress–strain curves of porcine soft tissue specimens. (**a**) Curves of skin specimens; (**b**) curves of subcutaneous specimens; (**c**) curves of muscle specimens; (**d**) curves of average stress–strain values of the four specimens for each type of tissue.

The complexity of multi-layered soft tissue compression behavior highlighted by this study underscores the challenges in standardizing the development and testing of dummy skin materials to mimic soft tissue properties. One limitation of this study is that factors such as age, gender, time of death, or storage methods were not controlled for the porcine soft tissue samples. To substantiate our findings, we conducted compression tests on various porcine soft tissue specimens obtained from pork meat purchased at different times from the supermarket. The specimen sizes were similar to those listed in Table 1, with some variations in thickness. We prepared four specimens of each type for skin, subcutaneous tissue, muscle, and multi-layer tissue. A loading rate of 0.5 mm/s was applied during the tests. The results are presented in Figure 9. Although the curve values in Figure 9 differ from those in Figure 3; the trends in all curves are consistent. The four skin curves in Figure 9a exhibit similar trends to the top two curves in Figure 3a. Therefore, it is reasonable to exclude the bottom curve in Figure 3a from further analysis. This specimen generally exhibits greater stiffness than the one shown in Figure 3. However, the similar trends in the curves lead to the same conclusion regarding the linear to nonlinear transition of deformation with increasing strain and the strain-hardening effect.

We compared the quasi-static transversely isotropic mechanical properties of soft tissue and car crush dummy skin based on the one-term Ogden and generalized Maxwell models. Active mechanical properties of muscles and anisotropic material properties will be included in our future studies. However, it is impractical to expect all mechanical properties of dummies to mirror those of humans. In the performance of comparison, an index may be utilized. For example, Yang [33,34] using specific energy absorption and collision efficiency as key indicators to compare the collision performance of various protection structures. Considering the application purpose of dummies, similar trends in mechanical response or energy absorption during crash test may also be the main aspects regarding the biofidelity of dummy skins. Therefore, energy related indices could potentially be devised for more effective crash test comparisons. It is important to note that both soft tissue and dummy skin are layered structures. Thanks to the power of simulation, the mechanical properties of dummy skin can be accurately estimated by fine—tuning the mechanical parameters of each individual layer. This not only aids in understanding the behavior of the materials but also lays the groundwork for more sophisticated analyses. A layered approach to modeling the mechanical properties of both the soft tissue and dummy skin particularly under dynamic loading conditions like those experienced in a car crash will be studied further in the future studies.

## 5. Conclusions

This study offers a comprehensive examination of the compressive mechanical properties of multi-layered soft tissues and car crash dummy skin material. The one-term hyperelastic Ogden model demonstrated a robust fit for the compressive data of both soft tissue and rubber material of the dummy skin samples when subjected to compressions up to 20% at loading rates below 1 mm/s. We presented the one-term Ogden hyperelastic models and generalized Maxwell viscoelastic models for single-layer and multi-layer soft tissues, as well as dummy skin materials. These models, along with our comparative analysis, illustrate the impact of layer structure and material composition on the compressive behavior of these complex biological tissues and bionic skin materials. Our results highlight that the mechanical response of soft tissue is significantly influenced by loading speed, a factor that current dummy skin materials fail to fully consider. Furthermore, dummy skin materials show shorter relaxation times and lower stress decay rates than soft tissues, indicating a less viscous characteristic. These findings are instrumental for the development of novel bionic skin materials or structures to more precisely replicate the biomechanical properties of soft tissues, thereby enhancing the accuracy and reliability of car crash test dummies.

## Figures and Tables

**Table 1:** Parameters of the soft tissue and dummy skin specimens.

Specimen (Length × Width × Thickness) mm		μ (kPa)	α	*e*
Soft tissue	Skin (50 × 50 × 7)	0.159	6.552	1.552 × 10^−7^
	Subcutaneous tissue (50 × 50 × 3)	0.049	6.421	3.984 × 10^−7^
	Muscle (51 × 51 × 17)	0.115	5.880	3.665 × 10^−7^
	multi-layer (51 × 51 × 35)	0.763	6.395	5.919 × 10^−7^
Dummy skin	Rubber (47 × 48 × 2)	2.029	5.424	9.520 × 10^−8^
	Sponge (46 × 44 × 40)	10.403	1.369	9.880 × 10^−7^
	multi-layer (50 × 53 × 17)	5.59131	4.008	1.220 × 10^−6^

**Table 2:** Dimension and Maxwell model parameters of the soft tissue and dummy skin from stress relaxation tests.

Specimen	Thickness (mm)	E_0_ (kPa)	E_1_ (kPa)	E_2_ (kPa)	τ_1_ (s)	τ_2_ (s)	*e*
Muscle	10.00	1.613	1.497	1.217	0.960	19.140	4.996 × 10^−7^
Subcu	18.50	4.195	1.686	1.579	1.285	21.660	2.140 × 10^−7^
Skin and subcu	17.50	0.628	0.331	0.234	0.963	17.693	9.302 × 10^−8^
Full tissue	40.06	9.618	4.248	4.909	1.559	26.317	2.053 × 10^−8^
Rubber	2.20	17.601	1.231	0.919	1.359	13.465	1.237 × 10^−8^
Sponge	40.00	9.082	0.613	0.943	2.488	26.264	1.558 × 10^−8^
Full dum. skin	17.00	21.922	2.188	2.559	1.816	22.743	8.890 × 10^−8^

**Table 3:** Viscoelastic characteristics of the dummy skin and soft tissue specimens.

	Skin	Subcutaneous Tissue	Muscle	Rubber	Sponge	Dummy Skin
σ_0_ (kPa)	1.59	6.70	13.91	22.16	10.72	28.71
σ_∞_ (kPa)	0.97	4.17	5.88	19.77	9.79	24.34
τ (s)	3.0	9.0	27.6	8.4	7.2	18
γ (%)	56.0	50.0	65.4	16.1	15.2	18.9

## Data Availability

The data underlying the results presented in this paper are available upon reasonable request.
